# Early identification of patients at risk of difficult intubation in the ICU: development and validation of the MACOCHA score in a multicenter cohort study

**DOI:** 10.1186/cc12095

**Published:** 2013-03-19

**Authors:** A De Jong, N Molinari, N Terzi, N Mongardon, B Jung, S Jaber

**Affiliations:** 1 Montpellier University Hospital, Montpellier, France; 2 Caen University Hospital, Caen, France; 3 Cochin University Hospital, Paris, France

## Introduction

Difficult intubation (DI) in the ICU is a challenging issue, associated with severe life-threatening complications [1,2]. The objective was to develop and validate a simplified score for identifying patients with DI in the ICU and to report related complications.

## Methods

Data collected in a prospective multicenter-study from 1,000 consecutive intubations from 42 ICUs were used to develop a simplified score of DI, which was then validated externally in 400 consecutive intubation procedures from 18 other ICUs and internally by bootstrap on 1,000 iterations.

## Results

In multivariate analysis, the main predictors of DI (incidence = 11.3%) were related to the patient (Mallampati score III or IV, obstructive sleep apnea syndrome, reduced mobility of cervical spine, limited mouth opening), to pathology (severe hypoxia, coma) and to the operator (non-anesthesiologist). From the β-parameter, a seven-item simplified score (MACOCHA score; Table 1) was built, with an area under the curve (AUC) of 0.89 (95% CI = 0.85 to 0.94). In the validation cohort (prevalence of DI = 8%), the AUC was of 0.86 (95% CI = 0.76 to 0.96), with a sensitivity of 73%, a specificity of 89%, a negative predictive value of 98% and a positive predictive value of 36%. After internal validation by bootstrap, the AUC was 0.89 (95% CI = 0.86 to 0.93). Severe life-threatening events (severe hypoxia, collapse, cardiac arrest or death) occurred in 38% of the 1,000 cases. Patients with DI (*n = *113) had significantly higher severe life-threatening complications than those who had a non-DI (51% vs. 36%, *P <*0.0001).

**Table 1:** MACOCHA score calculation worksheet

	Points
Factors related to patient	
Mallampati score III or IV	5
Obstructive apnea syndrome	2
Reduced mobility of cervical spine	1
Limited mouth opening <3 cm	1
Factors related to pathology	
Coma	1
Severe hypoxemia (<80%)	1
Factor related to operator	
Non-anaesthesiologist	1
Total	12

## Conclusion

DI in ICU is strongly associated with severe life-threatening complications. A simple score including seven clinical items discriminates difficult and non-DI in ICU.
